# Book Reviews

**Published:** 1805-05-01

**Authors:** 


### CRITICAL ANALYSIS
### OF THE
### RECENT PUBLICATIONS
### ON THE
### DIFFERENT BRANCHES OF PHYSIC, SURGERY, AND MEDICAL PHILOSOPHY.

---

*A Reply to Mr. Edlin's two Cases of Gout, said to have terminated in Death in consequence of the external Use of Ice and cold Water. To which is added, an Instance of the fatal Effects of encouraged Gout. With Observations, Cautions, &c.* By ROBERT KINGLAKE, M. D. Physician at Taunton. 8vo. pp. 61.

THE particulars of Mr. Baker's illness were contained in our account of Mr. Edlin's Two Cases. In THE REPLY, Dr. Kinglake first remarks, that though the ACCOUNT states two cases in its title, yet it contains only Mr. Baker's, the second being supported on no other authority than the unrecorded recollection of an event which was supposed to have happened thirty years ago, and for which there is no testimony of professional men. Mr. Edlin might as well have written *three cases*, as he brings forward the late Doctor Gregory's, which rests on still worse authority than the former. It is first stated by an anonymous writer; afterwards contradicted by Dr. Duncan, in the last volume of Med. Commentaries; and, lastly, half confirmed by a passage taken from a MS. copy of Dr. Saunders's Lectures, beginning thus. "As far as I can learn, cold was the cause of the late Dr. Gregory's death." Besides the caution used by Dr. Saunders, there is every reason to believe that this lecture was delivered before Dr. Duncan had published the true statement of Dr. Gregory's death.

Dr. Kinglake, in his remarks on the account given of Mr. Baker's death, observes, that it is deficient in making no mention of the previous previous state of his health; (some other objections follow, which we shall at present omit) that the absence of fever is inconsistent with the existence of gout; that, by Mr. Edlin's account, Mr. Baker was doubtful whether the disease was gout; and that we are uninformed whether his habit was gouty; that, however, his treatment of himself was not only judicious but efficacious, inasmuch as he found relief: Still Dr. K. inquires, why ice should have been applied, before it was ascertained that water, at the temperature of the spring, was found insufficient? Not that the practice is objected to; on the contrary, we are told, it appears, as was before observed, by the effect, to have been highly proper.

'Mr. Edlin found "that the pain was quite gone, that the patient considered himself in some measure as well, and that the pulse was at 78;" a situation enviably convalescent, and, surely, such as might justify rising from bed. But fainting occurred in the exertion; no unusual accident after a confinement of some days by acute pain, but very unlikely to have happened in this case, in the formidable manner stated, as the patient not long afterwards took breakfast, and soon after that was encouraged, by his own feelings, to combat an incipient inflammation on his knees with the same remedy.

'A man of liberality far superior to what Mr. Edlin has discovered in the present controversy, and one possessing much less questionable claims to implicit credit than he in my estimation does, could not readily persuade me that a patient who had incurred a serious fainting fit by the use of a new remedy, would so quickly return to the charge, and subject himself to the renewed injury of a noxious agent. This procedure bears a much less resemblance to human nature, than it does to the possible fiction of Mr. Edlin.'

After this follows a transcript of Mr. Edlin's history of the disease, from the time he was sent for at one o'clock in the afternoon, with the following remarks:

"This paragraph contains the recital of symptoms which have every appearance of being most extravagantly exaggerated. The account is only conceivable (in my apprehension at least) by considering it as a renewal of a fainting paroxysm, from what cause does not appear. No complaint is made of pain; palpitation of the heart and icy coldness of the stomach are not characteristic symptoms of a high degree of visceral excitement; they rather indicate a diminution of motive energy, and are not explicable by any rational idea of transferred irritation. But what says the patient in this gloomy narrative? — Not one word. Is he speechless?—Is he either epileptic, or apoplectic? It does not appear so. Did he not, from his medical familiarity with disease, from his own sensations, and his own particular knowledge of his general health, assign any probable cause for the alarming change that had occurred? Did he attribute it to the cooling treatment he had undergone, or did he attempt its vindication from such a charge?

charge? These important facts are all wrapped in mysterious and suspicious silence. It may be fairly presumed, nothing was said by the patient to the disadvantage of his treatment, or it would have too well suited Mr. Edlin's purpose for him to have withheld the statement of it.

"Dr. Haworth saw the case at too late a stage to form a correct opinion of its cause. Mr. Edlin, no doubt, furnished him with sufficient grounds to persuade him (according to the prevailing notions of gout) that it was an instance of that disease having been repelled from the extremities. But the professional concurrence of Dr. Haworth and Mr. Edlin is no adequate authority for admitting their opinion of the case to be correct. It was founded on the vulgar and erroneous notion of the nature of gout. Neither of them, with all their flippancy of decision, ever saw a case of repelled gout.

"They might, in proportion to their opportunities for observation, have seen many instances of the protracted pain of gout in the joints of the extremities, having propagated by sympathetic influence, fatal irritation to the system; but blinded by groundless prejudices, they could not perceive this scientific truth. It appears to me, through the mists of Mr. Edlin's partial statement, that the disastrous course of this case did not arise from any irremediable grievance of the heart and stomach, but that it was rather induced by the stimulant treatment to which the patient was subjected; for it is observed he was liberally dosed with æther, camphorated julep, and opiated confection, and farther overwhelmed with external heat; however, perspiration at length occurred, and produced by its *cooling* influence some relief.

"The consultation with Dr. Haworth, is stated to have terminated in an approval of Mr. Edlin's mode of treating the patient! Though "the skin was burning hot, the pulse at 96 strokes in a minute, the tongue dry and furred, and the respiration hurried,' the stimulant treatment is prosecuted, with an avowed view of driving the repelled gout, back to the extremities; but alas! the *sagacious* endeavour missed its aim, and instead of replacing the remedial disease on the intended parts, it probably demolished the very fabric of life, by kindling, throughout the system, an inflammatory degree of morbid temperature.

"However this violent re-action of the arterial system was excited, it was repugnant to the most obvious laws of organic motion in the animal economy, and to every rule of common sense, to attempt to calm it by adding to its force. By such heating treatment, the visceral parts (to use a fiery but applicable metaphor) must necessarily be burnt to death, before the extremities could be even scorched with the combustive violence.

"The appropriate treatment would have been a well ventilated room, sponging the '*burning skin*' with cold water, at short intervals, and copious dilution with cool aqueous liquids. Brandy, and the whole tribe of igneous stimulants, should have been withheld, held, and the patient might possibly have recovered, whilst under the circumstances of his mis-management it is almost inconceivable that the termination could have been different from what happened."

Such is the general outline of this important controversy, to which we have endeavoured to shew our accustomed impartiality. But our duty as public censors will not allow us to pass over some improprieties in the manner in which it has been conducted. Mr. Edlin begins his account, "A physician in one of our western counties has been amusing himself," &c. Afterwards, in his conversation with the deceased, he tells us, "I hinted that it was possible these (Dr. Kinglake's) cases might be fictitious, and that I was sorry to see him (Mr. Baker) misled by so shallow a performance." The reader will allow, with us, that it is no apology for such language, that, in another place, it is said, "In offering these observations to the world, I bear no personal ill will to Dr. Kinglake." The best thing we can say is, that Mr. Edlin could not be perfectly aware of the import of his own words.

We sincerely wish Dr. Kinglake had considered the thing in this point of view; but he has certainly shown, that he has not, at all times, a just command of temper. Whilst, with much propriety, he discants on Mr. Edlin's *hint*, he might have suggested, with more delicacy, that "such a suspicion could only arise in a mind capable of such artifices." Nor was it necessary to introduce Dr. Blegborough, merely to accuse him of interested motives in his opposition to the cold treatment of gout. Mr. Perry too might have been dismissed in much fewer words, and without ridicule: and the observation concerning the physician who was consulted, might have been spared. It would have been more candid, to have attended only to his practice, without regarding his opinion on the statement of Mr. Edlin: and if (which will perhaps admit of a doubt) "palpitation of the heart, and icy coldness of the stomach, are not characteristic symptoms of a high degree of visceral excitement, but rather indicate a diminution of motive energy," it will follow, that stimulating remedies might be proper to restore this energy.

In this remark, we wish only to encourage a proper degree of candour, as the means of divesting each other of passion, the great source of error in all controversies. We wish also that Dr. Kinglake should see, that though we do not think ourselves obliged to give any opinion concerning a practice much older than our own days, yet that, to us, there appears a deficiency in this part of his theory. Sickness, and a sensation of coldness, have occurred, previous to a gouty paroxysm, and have been relieved by it. They have also occurred on the sudden disappearance of the gouty inflammation.

On this, therefore, and on all other occasions, we wish to see less of figurative language in so important a branch of philosophy as medicine; and cannot help regretting, that, in the passage we have selected, selected, the author should consider the following as an "applicable metaphor:" "By such treatment the visceral parts must be burnt to death, before the extremities could be even scorched with combustive violence."

In some concluding observations, the author gives a case, in which the hot treatment of gout proved fatal. Though we have no doubt of the truth of every thing here stated; yet we wish, for the sake of the incredulous part of the public, that the name, at least, of the consulting physician had been added. We are aware this cannot always be done, from motives of delicacy to the surviving family of the deceased; but we regret the want of it exceedingly in this case.

Among his cautionary remarks, Dr. Kinglake observes, that 'the history of almost every medical improvement abounds with gross instances of illiberal opposition. The cooling treatment of the small pox, the proposal for alleviating the violence of that disease by inoculation; and, latterly, the further amendment of substituting for it the vaccine disorder, were severally resisted by dreadful denunciations; by the occasional production of disastrous cases; and by unrelenting personal invective against the benevolent advocates of the salutary recommendation.' This is all very just; and, as these practices have survived all this opposition, we doubt not, if Dr. Kinglake's improvement stands on as good ground, that it will maintain it as well as the others.

The Reply concludes with an earnest recommendation to the public, not to be alarmed by the publication of Mr. Baker's case; but to continue the use of cold water in all cases of gout. The practice, as we before remarked, is not new; but though discontinued, is not, on that account, to be supposed useless, or improper. The ancients, who had fewer remedies than we, made much greater use of baths, and with great success. The application of cold water in fevers is a very old remedy; though discontinued for a long series of years, till it was revived, with great success, by the physicians of our own days. In saying this, it is neither our wish to commend or condemn the remedy. Perhaps, like all others, there may be stages of the disease, or peculiarities of constitution, which would discourage even Dr. Kinglake from its use.

We wish much to know, whether any of our correspondents have tried the use of purgatives and bark, as recommended by Dr. Tavares, of Lisbon, in our Journal for August last.* By the doctor's experience on himself, as well as other numerous authorities, it appears not less successful than cold water, and is free from all the dangers which some apprehend from the latter.

---

* This Review was written before we received Dr. Lowrie's Communication, contained in our last Number.

*Observations*

*Observations addressed to the Public in general on the Cow-pox, showing that it originates in Scrofula, commonly called King's Evil, illustrated with Cases to prove that it is no Security against the Small-pox; also pointing out the dreadful Consequences of this new Disease so recently and rashly introduced into the Human Constitution; to which are added, Observations on the Small-pox Inoculation, proving it to be more beneficial to Society than the Vaccine. By* R. SQUIRRELL, M. D. formerly Resident Apothecary to the Small-pox and Inoculation Hospital. Octavo, pp. 75. London, 1805.

In the Introduction the Author expresses an apprehension that whatever he may say against the cow-pox may subject him to the imputation of prejudice, presumption, or conceit, and may be treated by some with ridicule and contempt. The latter, we conceive, the author has much the most reason to apprehend; but it does not become us to prejudge. Let us first trace the progress of his arguments in order.

They begin by stating Dr. Jenner's own account of the origin of the cow-pox, which, Dr. S. says, led him to suspect it was a species of scrofula. It would have been useful to hint how many species of scrofula there are, and how they are to be distinguished, or even to have said what he means by scrofula. This is followed by an analogical table of the opinion of farriers, respecting the grease, and of medical writers concerning scrofula. As the author quotes no authorities of farriers or physicians, it is not difficult to make the two diseases similar.

Next follow some remarks, indirectly addressed to parents; the principal object of which is to deter some from, and frighten others who have already adopted, vaccine inoculation. But before this it is remarked, that for any cause to produce scrofula the constitution must have a predisposition to that disease. Now this single argument oversets all the rest, for as a predisposition must be born with a person, consequently no inoculation can produce it. Various causes may indeed bring such a predisposition into action. Injuries of any kind, the long application of cold, and the presence of any local disease, are sufficient. Hence small-pox has produced it, more commonly what is called the natural small-pox, because in that mode the disease is most violent. Inoculation for the small-pox has however, in some cases, been sufficiently violent to induce scrofula in a predisposed constitution; and considering the immense number of vaccinated patients in so short a time, it is surprising so few, if any, fair cases can be produced in which scrofula has been excited. On the contrary, many well authenticated instances have occurred, in which scrofulous symptoms have disappeared in consequence of the new action excited by cow-pox. These remarks render it unnecessary that we should take notice of the author's proposed method of treatment to eradicate scrofula out of the constitution after it has been introduced by cow-pox.

 to

Suffice it to say, that, in general, we disapprove his plan, even supposing scrofula to exist from any cause.

After this follow a number of cases, most of which were first related in our Journal, have since been frequently canvassed, and have at length tended to convince our readers, that notwithstanding any trifling inconveniencies which may sometimes have attended this important discovery, they are as nothing, compared to what happen from small-pox inoculation. Cases of supposed small-pox after vaccination are taken indiscriminately from every source, and no notice is added that they have all been animadverted upon so as to satisfy every impartial mind. Lastly, the work concludes with a warm recommendation of small-pox inoculation, and a comparative view of its advantages above vaccination. In this view no notice is taken that cow-pox can only be spread by inoculation, whilst small-pox, being infectious in its effluvia, may, by the preservation of an individual, endanger a whole neighbourhood. At length the author conceiving he has completely silenced the advocates of vaccination, spends a great deal of time and paper in proving the advantages of small-pox under inoculation, when compared with the casual disease. As this has ceased to be a question for near forty years past, we should have been surprized to meet with it now, were it not that the rest of Dr. Squirrell's book surprized us still more.

---

*A Treatise on Lues Bovilla or Cow-pox.* By BENJAMIN MOSELEY, M.D. Physician to the Military Hospital at Chelsea, &c. 8vo. pp. 100. London, 1804.

WHEN we peruse the work before us, we are at a loss to know whether the author is in earnest or joke. In these gloomy times, *dulce est desipere;* but still, when we consider the importance of the subject, and the dignity which Dr. Moseley wishes to attach to his profession, we are very much in doubt whether the garrulity of age has any share in this sportive production.

Those who are accustomed to Dr. Moseley's writings must also be aware, that a display of learning makes no inconsiderable feature in most of them. Yet we were still surprized to find the small-pox traced as far back as Hippocrates. Who but Dr. Moseley would have thought that young Silenus, whom the father of the art describes as dying of a fever excited by intemperance and over-exercise, should have fallen a victim to small-pox? We perfectly agree with our facetious author, for we still think him in joke, that Silenus has as good, we cannot say a better claim, to small-pox than any other whose case and symptoms are described in either Galen or Hippocrates. Though the whole description of the disease is different from small-pox, excepting that there was fever and eruption, yet Dr. M. has no difficulty in accommodating all this. He describes as much of the case as suits himself; and when he finds this little is different from the small-pox, he has an admirable knack knack of throwing every thing into confusion. "In this case, says he, I have not detailed all the particulars, but I think enough appears to show it is not unreasonable to conclude that Silenus died of the small-pox. Hippocrates says, his πυρ, or burning fever, succeeded excesses committed by his patient, in bodily exertions and drinking; and this may account for the violence of the symptoms."

But Sydenham says, that in proportion as the symptoms are violent, the eruption is early; and here, in spite of hard drinking, the eruption was protracted to the eighth day. Alas! our English Hippocrates had never seen Africa, the American Isles, Constantinople, &c. *He* rarely quotes Greek; and when he ventures on Scripture, we never find him producing the Hebrew text. What should he know of things common in hot climates? Hear Dr. Moseley!

"The eruptions being retarded till the eighth day, or until a little before death (three days are not a little out of eleven) is not uncommon in hot climates, when life can be protracted; in cases where the blood has broken down its boundaries, and runs off by stool and urine, or by uterine discharges; *with all the functions of nature in confusion.*" Now if we are to select cases which resemble each other when *all is in confusion;* that is, when a disease loses its true character, we need never be at a loss to find small-pox, or any disease we wish for.

But it was necessary to find small-pox in Hippocrates; and indeed, in our opinion, it should have been traced as far back as Moses, in order to confirm our author's theory. "The small-pox, says he, is an atmospheric disease. It appears and disappears like other epidemics." "Atmospheric diseases, whether contagious or not, which rage epidemically, are not to be put a stop to, until the inscrutable cause which feeds their rage is exhausted." But we would ask if small-pox, like yellow fever, influenza, and some other epidemic diseases, depends only on the peculiar state of the atmosphere, without the necessity of any specific contagion, how happens it, that many towns have, by prudent precautions, preserved themselves free from variolous invasion for so many years? And how happens it that, since inoculation for small-pox has been introduced, the disease is constantly kept up in London?

To be short, the theory our author labours to maintain is, that cow-pox, like some other morbid poisons, produces what he calls a febrile commotion in the lymphatic system. Let us give his own words:

"Excite a febrile commotion in the lymphatic system, and saturate it with cow-pox virus—and that will, for a time, keep out the small-pox."

"The more the lymphatic system is thus acted on, the greater and longer the security against the small-pox."

If there is any meaning in this, it must be, that, as soon as the fever from cow-pox is over, the constitution will be susceptible of

 small-pox, small-pox; and if no fever is excited, nor any glandular affection, the constitution will never lose its susceptibility of small-pox. The contrary of all this has been in so many instances proved, that we wish our author would write in a graver mode, and explain his real meaning. At present we cannot be angry with one who entertains us so well; and the extreme good nature with which his pamphlet closes, makes us wish less exceptionable language had been chosen in comparing the Inventor of Vaccination with his immediate followers.

"Unfortunately for Dr. Jenner and his discovery," says our author, "he was not left to prosecute it deliberately in the country, and to investigate it in a quiet, philosophic manner, through a succession of many experimental years. The *manufacture* was still in its *embryo* (an odd state for a manufactory) when the raw materials were brought, unfit for use, to market; and they were snatched from his hands in their crude state by a set of medical jugglers, besotted and stupified with the gigantic novelty, and scattered like firebrands among the Philistines."

In our opinion, Dr. Jenner had completed his experiment sufficiently to make it known to the public; but we sincerely wish, with our author, that all his followers had shown the same honest investigation, the same temper, and the same caution, in their communications, as have distinguished the inventor.

---

*An Examination of that Part of the Evidence relative to Cow-pox, which was delivered to the Committee of the House of Commons by Two of the Surgeons of St. Thomas's Hospital, with some Remarks on inoculated Small-pox.* By W. R. Rogers; *illustrated with a coloured engraving.* Octavo, pp. 30. London, 1805.

By an Epistle Dedicatory, we find that Mr. Rogers is of the Herefordshire Militia; that he was formerly a pupil of Mr. Birch, and gathered most of his information from conversing with his teacher. We are ready to make large allowances for this attachment to preceptors, which always argues a grateful mind; but there is a time when men should think for themselves. However, we ought to add further, that this work is more free from personalities than most which have appeared on the subject; and if we make allowance for the frequency with which the author must have heard the question discussed on the side he has adopted, we may consider his production as the offspring of an honest, though, in our opinion, mistaken zeal.

The cases on which Messrs. Cline and Birch differed, were happily such as both agree in as to matter of fact, viz. that persons who have received the small-pox infection, and are inoculated with cow-pox before the eruption appears, are not secured from the first disease. Mr. Birch adds, that under similar circumstances the small-pox inoculation produces all the effects that are expected from it, as perfectly as when the patient has not been previously exposed to small-pox infection. As this is a subject that has often occurred, occurred, and not been sufficiently discussed, we shall offer a few remarks before we proceed any further.

It has been long since remarked, that the period between the reception of the small-pox virus by effluvia, and the commencement of the fever is usually a medium of twelve days. That, on the contrary, the period between inoculation of small-pox and the commencement of fever is a medium of eight or nine days. Consequently, if matter is inserted three, four, and in some instances five days after infectious effluvia has been inhaled, the constitution may still have the advantage derived from inoculation, as the operation may supersede the effect of the disease from effluvia. It will at once occur, that it is difficult always to ascertain this fact, because in some cases the casual small-pox is as mild as the inoculated, and in others the inoculated shows all the severity of the casual. However, the facts which the diligence of different inoculators have accumulated are sufficient to establish the law.

In the cow-pox the question is more easily decided, because when eruptions follow, it is easy to ascertain whether they are variolous or vaccine, consequently we are never at a loss to ascertain whether vaccination has superseded a previous exposure to variolous effluvia.

Hitherto the parties seem to agree in one main point, namely, that if a subject has been so far exposed to the effluvia of small-pox as to be infected by it, the consequence of that infection has not been superseded by inoculation from cow-pox. Mr. Birch adds, that inoculation from small-pox, undersimilar circumstances, would have superseded the effects by infection. Mr. Cline does not seem, by the evidence here stated, to have marked that difference with so much decision; and the reason will presently appear.

In all those cases in which inoculation for cow-pox was performed early enough to supersede the effect of effluvia from small-pox, the patient had the latter disease milder. This makes it difficult to say that the inoculation by cow-pox was altogether inefficacious. This uncertainty is the greater, because, in one instance, small-pox appeared only four days after inoculation for cow-pox. Consequently, the child must have received the small-pox effluvia eight days before vaccination, a period at which *small-pox* inoculation would have proved no security. In this case the disease was very severe. It is probably for this reason that Mr. Cline was too cautious to venture on a comparison between the effects of small-pox inoculation and vaccination, under circumstances of previous exposure; and it is much to be regretted that this important question is not yet solved.

Another very striking difference between the examination of these two gentlemen is, that Mr. Birch gives no dates, and deposes that he kept no notes. Those who are at all accustomed to watching the progress of morbid poisons, must be aware how dangerous it is to trust to the memory, even in more minute circumstances; but when this deficiency extends to an ignorance of the days of expo-

 sure, sure, inoculation, and eruption, the whole evidence amounts to nothing, because there is not a single foundation on which reasoning, much less inference, may rest.

After concluding the account of these examinations, our author informs us of 'Mr. Birch having taught his pupils the maxim, that experience was preferable to experiment.' We should have been at a loss to know the difference, had not the conclusion of the paragraph illustrated it. By this we find that it is better to avail ourselves of others' experiments than to perform them ourselves. This observation is in a certain degree just, because where we take the experiments of others, our own fidelity cannot be suspected. But under such circumstances we should either report the result as we receive it, or attend closely to the whole process, and make our own minutes with exactness. Neither of these were accomplished by Mr. Birch. He declined making the experiment, and trusted to his memory for the results. His evidence is therefore defective, and his *pupils* must learn better *maxims*, or how to apply them better. However, from these imperfect materials, Mr. Birch undertakes to draw conclusions which the premises, if better ascertained, would not infer. "Now, says Mr. Rogers, as it is agreed on all hands that the inoculation of small-pox, under similar circumstances, would supersede and destroy the infection naturally received, Mr. Birch took his stand on this ground, and has ever since steadily and firmly maintained, that on this account he was satisfied the experiment would not produce the results expected from it."

But fair induction, according to the common forms of logic, would never support Mr. Birch on such ground; for if we admit his facts, it will only follow that there is some difference not sufficiently understood between small-pox and vaccination, so that when a person has been exposed to the effluvia of the former, it may be safer to inoculate with small-pox, as a better ascertained means of superseding the effects of the casual infection. Mr. Cline, with his usual modest caution, leaves the question in doubt, how far the subsequent small-pox is not superseded by vaccination, but gives his opinion that the future disease is mitigated; "yet, adds our author, in the case of Mary Solloway, if I rightly understand him, Mr. C. states *the disease to have been very severe, but that the child recovered.*" No, Mr. Rogers, you do not rightly understand him, for you will find that the eruption appeared in four days after vaccination. In this case therefore the effluvia had been too long received for even *small-pox* inoculation to supercede its effects.

The author next examines the three positions admitted by the committee. The first is, that vaccination never proves fatal. The second, that it excites no new disorders. The third, that it is a perfect security against small-pox, and, in the end, likely to exterminate the disease.

In answer to the first, the author produces four cases. As these are all well known, we shall only express our astonishment that no more can be produced, considering the number of persons vaccinated, nated, and the great uncertainty of the effect of even a common puncture in some particular constitutions, or under some peculiar circumstances. Two of these cases have appeared before the public, in a variety of forms; the last is quoted from Dr. Squirrell, author of a Treatise on Indigestion, and vender of medicines.

The next position our author attempts to prove is, that cow-pox does, in some instances, excite a new disease different from itself. First, he says, it produces eruptions on the face and in other parts of the body. This is undeniable; but these eruptions are temporary, and are generally the effect of some previous inflammation, or breach of the skin, and much more frequently the new disease supersedes former eruptions, which have been of long standing.

2d. " A hasty abcess, which contains a fluid dissimilar to any other."—We shall be glad of further information on the subject of this hasty abcess, and its contents.

3d. " Glandular enlargements of the skin ; at first the size of a pea, then growing knotty and hard, at length suppurating." In confirmation of this, we are referred to two cases ; one of Rebecca Latchfield, daughter of a workman of Mr. Banks, in the Strand ; the other, a child of a servant of Mr. East, in the Adelphi. We have before now heard of the head-dress of Pontius Pilate's wife's chamber-maid ; and a reference to the daughter of a workman of Mr. Banks, Strand, and the child of a servant of Mr. East, Adelphi, reminded us of the venerable relic of a waiting-maid of that illustrious lady. If our author had given us his own authority, we should have been less anxious to satisfy ourselves as to the event of these cases, of which we have no information. As it is, our readers, who are anxious to know, will not require of us to advertise for the children of Mr. Banks's workman, or Mr. East's servant.

In the last place, continues our author, " with grief, (but confidently) I assert, that the great advantage which mankind were to have received from this discovery has not been attained ; from its being no security in numerous instances, against the infection of the natural small-pox."

The *numerous* cases adduced by our author, amount to the few which have been repeatedly before the public. We doubt not more have occurred : for, when we consider the immense extent of vaccination, and that the same event has occurred after small-pox, both in the casual way and under inoculation, we cannot expect that cow-pox should be a better security. That there were instances of small-pox occurring a second time in the same subject, most of the different medical collections may prove. Dr. Adams, in his " Morbid Poisons," in which he has shewn so much industry in collecting facts from all quarters, and which was published before vaccination was introduced, not only admits the possibility of small-pox occurring twice in the same subject, but expressly adds, that he conceives a certain space must intervene before the susceptibility to the disease can be restored, (Morb. Poisons, page 55.

Mr. Rogers concludes with some remarks on the advantages of small-pox inoculation. It is not difficult to prove these when every unfavourable incident that has occurred under vaccination is dwelt upon with so much exactness, and inoculation after two years of age is said to be *almost* unattended with danger. But we are surprised to hear the author speak of *feelings* on this occasion. Where are the feelings that would voluntarily subject an infant, though more than two years old, to the fever certain on small-pox inoculation? Even if anxiety for greater safety should induce this, where are the feelings that would selfishly expose a whole neighbourhood to the danger of so dreadful a disease? Baron Dimsdale, whose name is brought forward on this occasion by our author, it is well known, advised general inoculation as a preventive of the spreading of the disease; and we cannot help thinking that the legislature would now be justified in appointing seasons, when notice should be given of permission for general inoculation for small-pox, as long as any part of the public retains its predilection for that disease.

---

### *Dr. Jackson, on the Treatment of Fevers.*

( Continued from pp. 275—282. )

" There scarcely occurs an instance, where the febrile irritation obtains, in a perfectly equal degree, in every part of the system, at the same time. In one class the irritation is general,—the principal an l leading circumstance,—the local affections are secondary and subordinate. In another class, the local derangement seems to be primary, at least it is principal; it demands and obtains the principal attention in arranging the plan of cure. In acute diseases, where inflammation of internal organs is a prominent or leading feature, bleeding is the first and almost the only remedy. In real inflammation of the lungs, and parts in the cavity of the thorax, (the most frequent and the most serious of local inflammation,) bleeding possesses a decided power of doing good; but, in order to be effectual, it often requires to be carried to an extent, which common opinion, in the present times, regards with horror. It is not possible, by any previous examination, to determine precisely the quantity of blood, which ought to be taken away in inflammation of the lungs and surrounding parts. The measure can only be determined by effect, as it arises under the operation. It is a constant rule never to be forgotten, that as an effect, that is, relief from certain destructive symptoms, is the object of the practice adopted; so that object must be ensured before the means are dismissed. The quantity of two pounds of blood, in real inflammation of the lungs, is not excessive; it is not alwass sufficient; it may even be carried beyond three, without danger to life; nay, the object in many cases is not attained, till actual fainting, or a strong disposition to faint, is induced.

" Where real inflammation exists, bleeding is an indispensable remedy; but the existence of such condition is not always easily ascertained. It is apt to be confounded with pituitous suffocation, denominated peripneumonia notha; or with affections of respiration,—a fluctuating symptom of contagious fever. The distinction may often be known by actual inspection; it is difficult to detail the discriminating marks so precisely in description, as to preclude all ambiguity. — The real inflammation of the lungs, and parts within the cavity of the thorax, is generally attended with great tumult in the circulating system; but the expression of the fever is not always open; it is often, as it were, suffocated and oppressed; the heat is deep, strong and ardent, particularly on the chest; the pulse is usually frequent, but not uniformly so;—it is hurried, irregular, impeded in its movement,—without freedom or expansion; the heart sometimes beats, or throbs irregularly; and the whole organs concerned in circulation evidently labour, even struggle in the performance of their offices; the head-ach is frequently severe and rending, as if the skull were ready to split; sometimes it is heavy and oppressive; the eye is full and painful, the eye-ball distended, — glistening rather than inflamed; the countenance is flushed, clouded,—often grim and expressive of pain,—or dark and bloated; the respiration is hurried,—even to panting; the breath is hot; the lips dry,—with intense thirst; the cough is troublesome, dry, or without expectoration; the pains in the chest and sides are more or less severe;—sometimes sharp and stinging—aggravated by motion; sometimes obscure, dull, with a sense of oppression and difficulty of expanding the chest; the back and limbs ache severely, as if all the moving parts were broken and disjointed; together with these, there are particular sensations of heavy distress,—a sense of fulness, and a suspension of natural secretions. Such form of disease is more common in cold weather than at other times; and its origin is often traced to sudden or long exposure to cold. —When the symptoms described are present, blood must be taken away without loss of time. It is adviseable, that it flow from a large orifice; and that it be allowed to flow till the symptoms are completely removed; till the chest can be filled with ease and freedom; till sweat and relaxation of excretories take place; in short, till the existing movement be completely subverted. The foundation of cure is then laid, and the course of the disease is short.

"Bleeding is thus a powerful means in the cure of acute diseases; capable of a great deal of good, or a great deal of harm, according to the circumstances under which it is applied, and the mode in which it is managed. It arrests perverted movement, or it increases susceptibility to impression; in consequence of which, other remedies become capable of producing those motions, which are analagous to, or which are the identical movements of health. In this view, it is useful in the commencement of fever. If indeed the circumstances, in which it is employed, be the proper ones, and the management of the remedy be well conducted, it it seldom fails of producing a salutary effect. In the advanced stages of acute disease, it is only useful occasionally; for its effects are only decided, where the movements are not yet rooted, that is, where they do not yet tend, by a regular though diseased process, to an issue, which terminates the course of the derangement. In such case, it is subordinate; in the beginning, in some forms of disease, it may be said to be sovereign.

"In the emetic class of remedies is found another of the powerful aids employed by medical men, in arresting the perverted rhythm of organic movement, and in restoring susceptibility of impression, preparatory to the application of other means. The emetics of the antimonial class act powerfully, and, if properly managed, produce great effect upon animal bodies. They are often employed in the early stages of fever; and in some conditions and stages of fever, their benefits are signal. The lancet commands the cure of a certain form of fever, the action of which is manifested in the grosser circle of circulation; emetics have great power, where the action is directed to organs of secretion,—not where the functions are suspected, but where the process is in some manner changed; more particularly, where the diseased organ is connected with the part upon which the action of the emetic has its first and immediate operation. But though emetics are eminently useful, they are not indiscriminately useful. The conditions which define their use, require to be well and minutely considered:—the present view is only an outline. In the first form of fever, that is, the plethoric, where the action is so particularly manifested in the grosser circle of circulation, and where the advantages of bleeding are so obvious; a form of disease frequent in spring, in dry weather, and among Europeans soon after their arrival in tropical climates, the use of emetics is not beneficial, not proper, perhaps not safe, without previous preparation. On the contrary, where there are congestions in secreting organs, or rather excess of secretions, as of bile and mucus, their benefits are great and evident. They are then commonly employed; and they are employed with manifest advantage, in intermittents, remittents, and autumnal bilious fevers, in diarrhœa and dysentery, in most forms of catarrhal affection, or defluxion on the lungs, in epidemic or erysipelatous sore throat, in eruptive fevers, and generally in specific contagions. In the artificial contagious fever, the cause of which is generated in crouded places, and the action of which has its first effect upon the stomach and bowels, an emetic is the first, as it is the peculiar remedy. It often alone removes the disease in the beginning. Even after the febrile irritation has become general, if the process be not far advanced in progress, so as to be engrafted in the action of the system, an emetic, followed by warm and cold bathing, and that followed by the application of blisters to the forehead, temples, neck, or between the shoulders, frequently cuts it short; at least, perfectly removes the danger, which might be expected to ensue from the continuance of it. But while emetics are useful in congestions gestions connected with increased quantity and changed condition in the secretions, they are not useful in congestions in the actually circulating system, that is, in inflammation of parts or organs, no more than they are in general fever connected with plethora and accompanied with suspension of natural secretions.

It would require much detail, to identify all the circumstances and conditions, and to estimate correctly all the advantages or disadvantages which result from the use of emetics; but it may be considered as a general rule, and, it is believed, it is a safe one, that emetics have no place where the action of the disease is chiefly manifested in the circle of the greater circulation, with suspended secretion, the case in which the lancet is so useful. On the contrary, where the action of the disease is manifested in the secretions of secreting organs, in which case bleeding is rarely necessary, the benefits of emetics are remarkable. Besides a previous and correct estimate of the conditions, which prescribe the use of emetics in acute diseases; the manner of exhibiting the remedy, and of managing the patient while under its operation, deserves some consideration, in order to ensure fully the beneficial effect. In diseases of the intermitting, remitting, and bilious forms, the antimonial emetics obtain the preference, as they ought in all cases, where strong and general effect is required. It is necessary, in the exhibition of emetics, that the state of the stomach, if not naturally, be rendered artificially susceptible of the full effect of the operation. In diseases, with an increase of the bilious secretion, or in constitutions which naturally possess the bilious character, the operation of emetics is generally full and effective, and the benefits are in proportion. In accidental conditions of disease, or in constitutional circumstances of subject, where this character is wanting, or where the excess of secretions tend to phlegm, the stomach is acted upon with difficulty, and the effect of the operation is then seldom complete. In such case it is adviseable to give some water, as hot as it can be drank, either alone, or with the addition of an alkaline salt, previous to the exhibition of the emetic. It is farther adviseable, that persons, under the operation of emetics, be placed in bed, well covered with clothes, and that the air of the apartment be rather of a high temperature. It is also serviceable in many cases, as aiding the effectual operation, that the feet be previously bathed in warm water, or that the limbs be fomented with cloths wrung out of hot water; in short, that the general sensibility to impression be increased, as well as that the condition of the stomach itself be consulted, and previously disposed to answer effectually in operation."

[ To be continued. ]

Account

